# Supplementary material for: Quasiperiodic rhythms of the inferior olive
Source: PLoS Comput Biol. 2019 May 6;15(5):e1006475. doi: 10.1371/journal.pcbi.1006475 (PMC6538185; doi:10.1371/journal.pcbi.1006475)
Supplement: S1 Fig — The autocorrelogram of the same Purkinje cell as shown in Fig 2A (10 ms bins) convolved with a 5 ms kernel (red curve) compared to the convolved inter-complex spike interval (ICSI) histogram (orange curve). The auto-correlogram includes also intervals between non-consecutive complex spikes. Consequently, on a short time scale, both auto-correlogram and ICSI histogram are identical, but they diverge at longer time scales. (PDF) [file pcbi.1006475.s001.pdf]

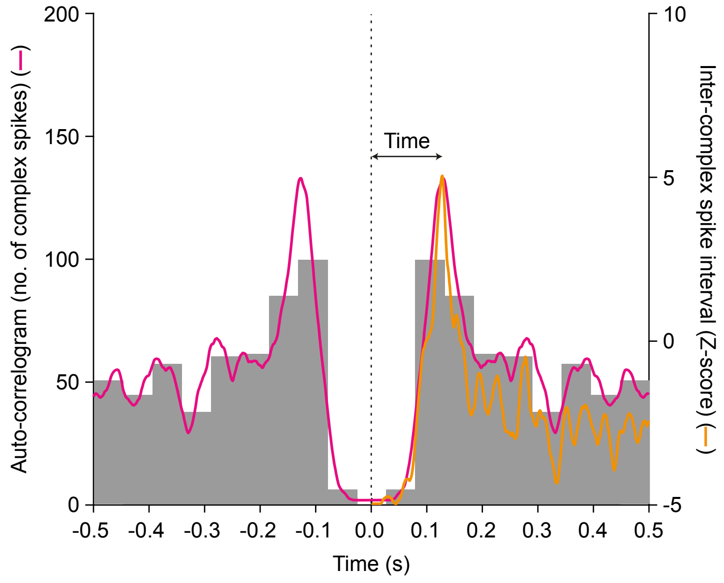

Fig. S1 | Comparison of autocorrelogram with inter-complex spike interval histogram.

The autocorrelogram of the same Purkinje cell as shown in Fig. 2A (10 ms bins) convolved with a 5 ms kernel (red curve) compared to the convolved inter-complex spike interval (ICSI) histogram (orange curve). The auto-correlogram includes also intervals between non-consecutive complex spikes. Consequently, on a short time scale, both auto-correlogram and ICSI histogram are identical, but they diverge at longer time scales.
