# Supplementary material for: Quasiperiodic rhythms of the inferior olive
Source: PLoS Comput Biol. 2019 May 6;15(5):e1006475. doi: 10.1371/journal.pcbi.1006475 (PMC6538185; doi:10.1371/journal.pcbi.1006475)
Supplement: S2 Fig — (A) Mice received air puff stimulation of their whisker pad. (B) Purkinje cell recordings during sensory stimulation revealed that most trials had either no, one or two complex spikes during the 200 ms after stimulus onset. We separated the trials according to this classification. Note that for the trials with a single complex spike, we considered the trials with a complex spike during the first 100 ms, but not during between 100 and 200 ms. (C) Averaged whisker traces (based on n = 35 Purkinje cells) show a reflexive whisker movement triggered by the air puff, consisting of an initial backward movement (largely caused directly by the air flow) followed by an active protraction. Trials in which a complex spike was detected only during the first 100 ms after the stimulus (blue line) had on average a slightly larger protraction than the trials without a complex spike (black line). The trials with two complex spikes also had a stronger protraction than the trials without a complex spike, but showed in addition a more protracted position later on during the trial (red arrow and red line). (D) Averaged subtracted traces showing the differences between trials with, respectively, a single complex spike (blue line) and two complex spikes (red line) and the trials without a complex spike. The occurrence of recurrent complex spike firing was thus reflected in the behavior of the mice. Shaded areas indicate the SEM. * p < 0.05; *** p < 0.001; Wilcoxon match-pairs test after Benjamini-Hochberg correction for multiple comparisons. (PDF) [file pcbi.1006475.s002.pdf]

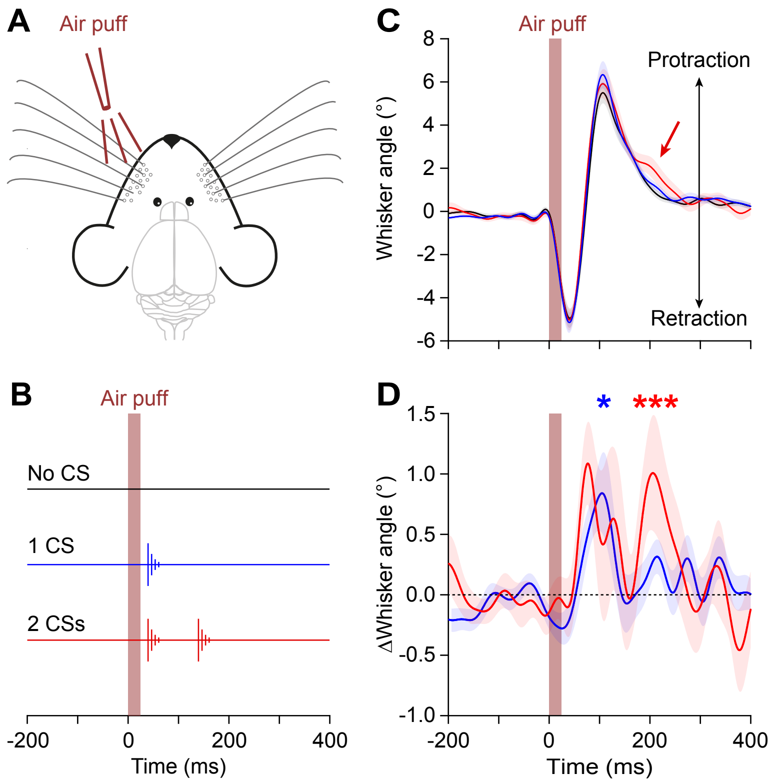

Fig. S2 | Complex spikes correlate with whisker protraction.
(A) Mice received air puff stimulation of their whisker pad. **(B)** Purkinje cell recordings during sensory stimulation revealed that most trials had either no, one or two complex spikes during the 200 ms after stimulus onset. We separated the trials according to this classification. Note that for the trials with a single complex spike, we considered the trials with a complex spike during the first 100 ms, but not during between 100 and 200 ms. **(C)** Averaged whisker traces (based on n = 35 Purkinje cells) show a reflexive whisker movement triggered by the air puff, consisting of an initial backward movement (largely caused directly by the air flow) followed by an active protraction. Trials in which a complex spike was detected only during the first 100 ms after the stimulus (blue line) had on average a slightly larger protraction than the trials without a complex spike (black line). The trials with two complex spikes also had a stronger protraction than the trials without a complex spike, but showed in addition a more protracted position later on during the trial (red arrow and red line). **(D)** Averaged subtracted traces showing the differences between trials with, respectively, a single complex spike (blue line) and two complex spikes (red line) and the trials without a complex spike. The occurrence of recurrent complex spike firing was thus reflected in the behavior of the mice. Shaded areas indicate the SEM. * $p < 0.05$; *** $p < 0.001$; Wilcoxon match-pairs test after Benjamini-Hochberg correction for multiple comparisons.
