# Supplementary material for: Quasiperiodic rhythms of the inferior olive
Source: PLoS Comput Biol. 2019 May 6;15(5):e1006475. doi: 10.1371/journal.pcbi.1006475 (PMC6538185; doi:10.1371/journal.pcbi.1006475)
Supplement: S3 Fig — The inferior olivary model has, as the inferior olive itself, boundaries. The impact of boundaries in connectivity of cells in the in vivo data (and by extension on the current leak through gap junctions) is, however, not known. The algorithm that generates connectivity enforces mean connectivity across cells, which increases the degree of clustering along the edges, but has at most a mild impact on the current leak through gap junctions. It is likely that the extra clustering degree along the edges may lead to a mild increase of coherence in STOs between neighbors, though this should not affect the overall conclusion–that the phase-dependency of the STO under the presence of noise is at most short-lived. The data are represented as stacked bar plots. All data were tested using Kolmogorov-Smirnov tests. (PDF) [file pcbi.1006475.s003.pdf]

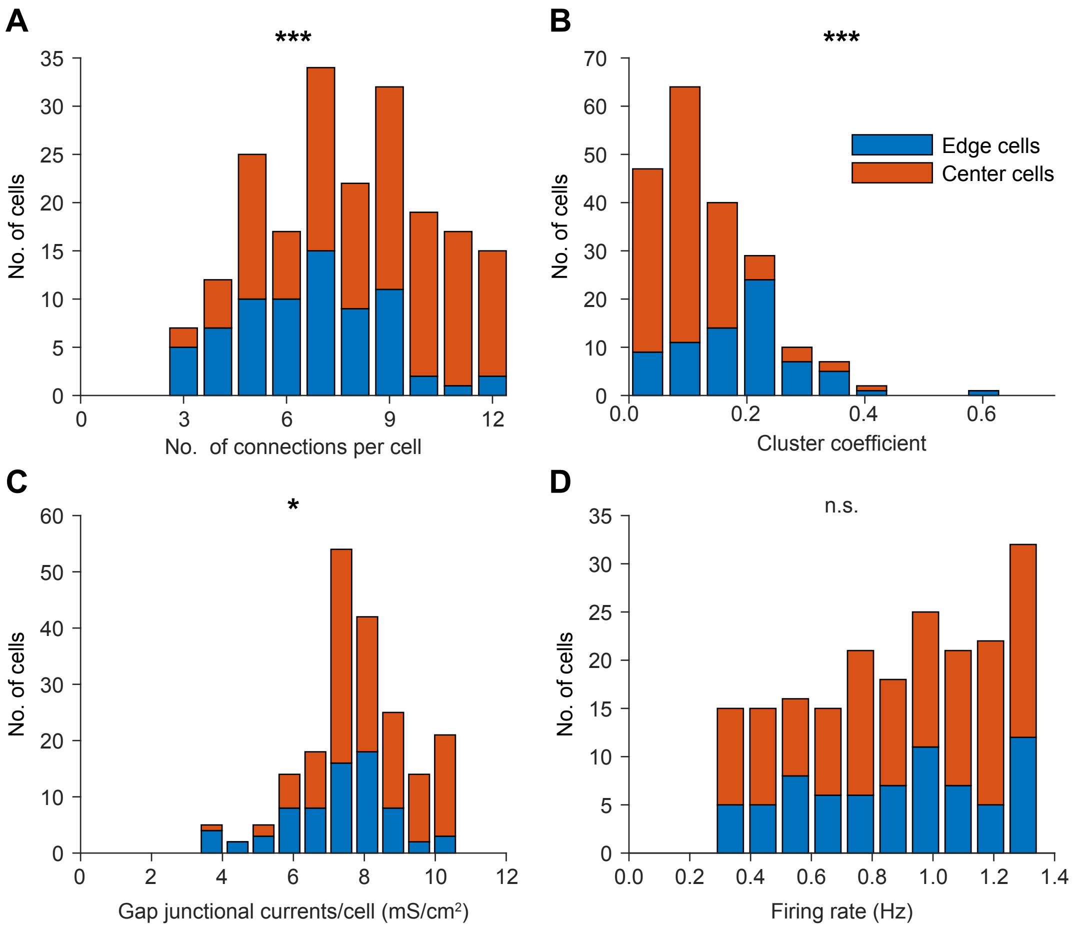


**Fig. S3 | Behavior of cells on the edges of the model.**
The inferior olivary model has, as the inferior olive itself, boundaries. The impact of boundaries in connectivity of cells in the *in vivo* data (and by extension on the current leak through gap junctions) is, however, not known. The algorithm that generates connectivity enforces mean connectivity across cells, which increases the degree of clustering along the edges, but has at most a mild impact on the current leak through gap junctions. It is likely that the extra clustering degree along the edges may lead to a mild increase of coherence in STOs between neighbors, though this should not affect the overall conclusion - that the phase dependency of the STO under the presence of noise is at most short-lived. The data are represented as stacked bar plots. All data were tested using Kolmogorov-Smirnov tests.
