# Supplementary material for: Quasiperiodic rhythms of the inferior olive
Source: PLoS Comput Biol. 2019 May 6;15(5):e1006475. doi: 10.1371/journal.pcbi.1006475 (PMC6538185; doi:10.1371/journal.pcbi.1006475)
Supplement: S4 Fig — (A) Baseline input was chosen such that the cells of the model networks would produce approximately 1 Hz of spontaneous firing rate. The absence of gap junctional coupling in “mutant” model networks (B) leads to increased firing rate, which was compensated for by increasing the leak current of the membrane by 0.003 mS/cm2. (PDF) [file pcbi.1006475.s004.pdf]

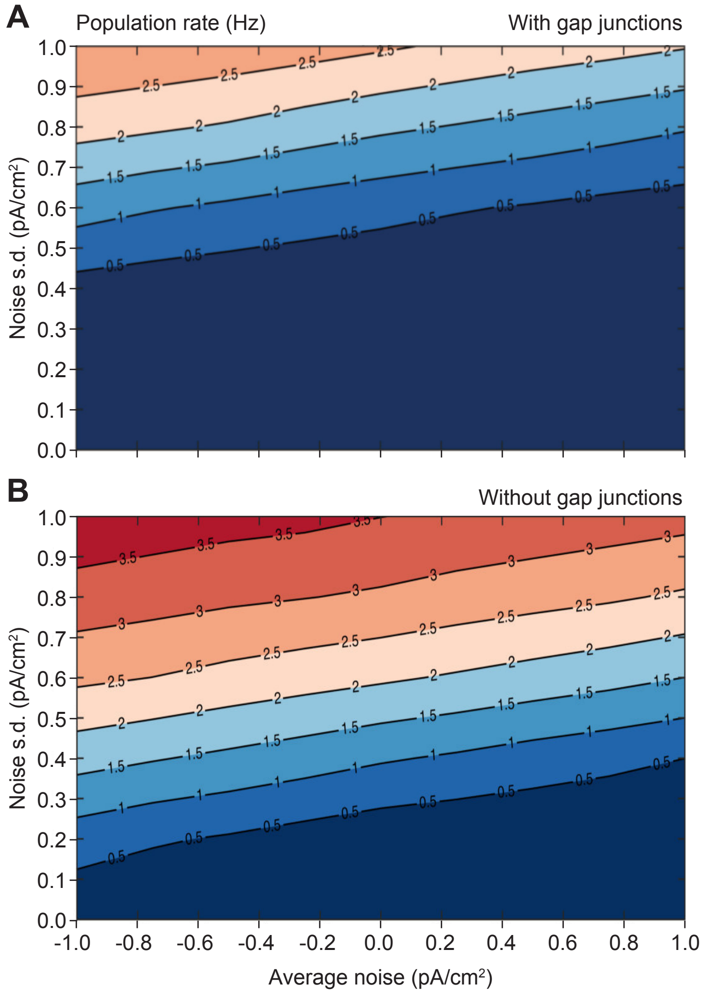

Fig. S4 | Contour plot indicating the average firing rate of networks as a function of the mean and standard deviation of the OU input.
(A) Baseline input was chosen such that the cells of the model networks would produce approximately 1 Hz of spontaneous firing rate. The absence of gap junctional coupling in “mutant” model networks (**B**) leads to increased firing rate, which was compensated for by increasing the leak current of the membrane by 0.003 mS/cm^2 (data not shown).
