# Supplementary material for: Quasiperiodic rhythms of the inferior olive
Source: PLoS Comput Biol. 2019 May 6;15(5):e1006475. doi: 10.1371/journal.pcbi.1006475 (PMC6538185; doi:10.1371/journal.pcbi.1006475)
Supplement: S5 Fig — Model networks have been computed using a systematic exploration of the parameter space using 56 instances of the network model. The contour plots indicate isolines for synchrony (A), frequency of subthreshold oscillations (STOs), population firing rate and proportion of active cells (B). The results presented in the main text come from a model network with parameters chosen such as to display STOs with mean of 9 Hz, a population rate of about 1 Hz and with more than 70% of cells firing in three seconds (95% of cells fire within 10 s of simulation time). The position of this network in the contour plots is indicated with a red circle. In A, the thumbnails exemplify behaviors of extreme instances of the model network both as membrane potentials traces (top) and as heatmaps of the membrane potential (bottom). Arrows indicate parameter space coordinates of these examples. The decay of the Ornstein-Uhlenbeck process (τ) mostly impacts the firing rate of the model networks, while noise correlation (η) has a direct effect on synchrony. (B) Gap junctions amplify the input correlation given to the neurons, while having a minor effect on other aspects of the network dynamics. (PDF) [file pcbi.1006475.s005.pdf]

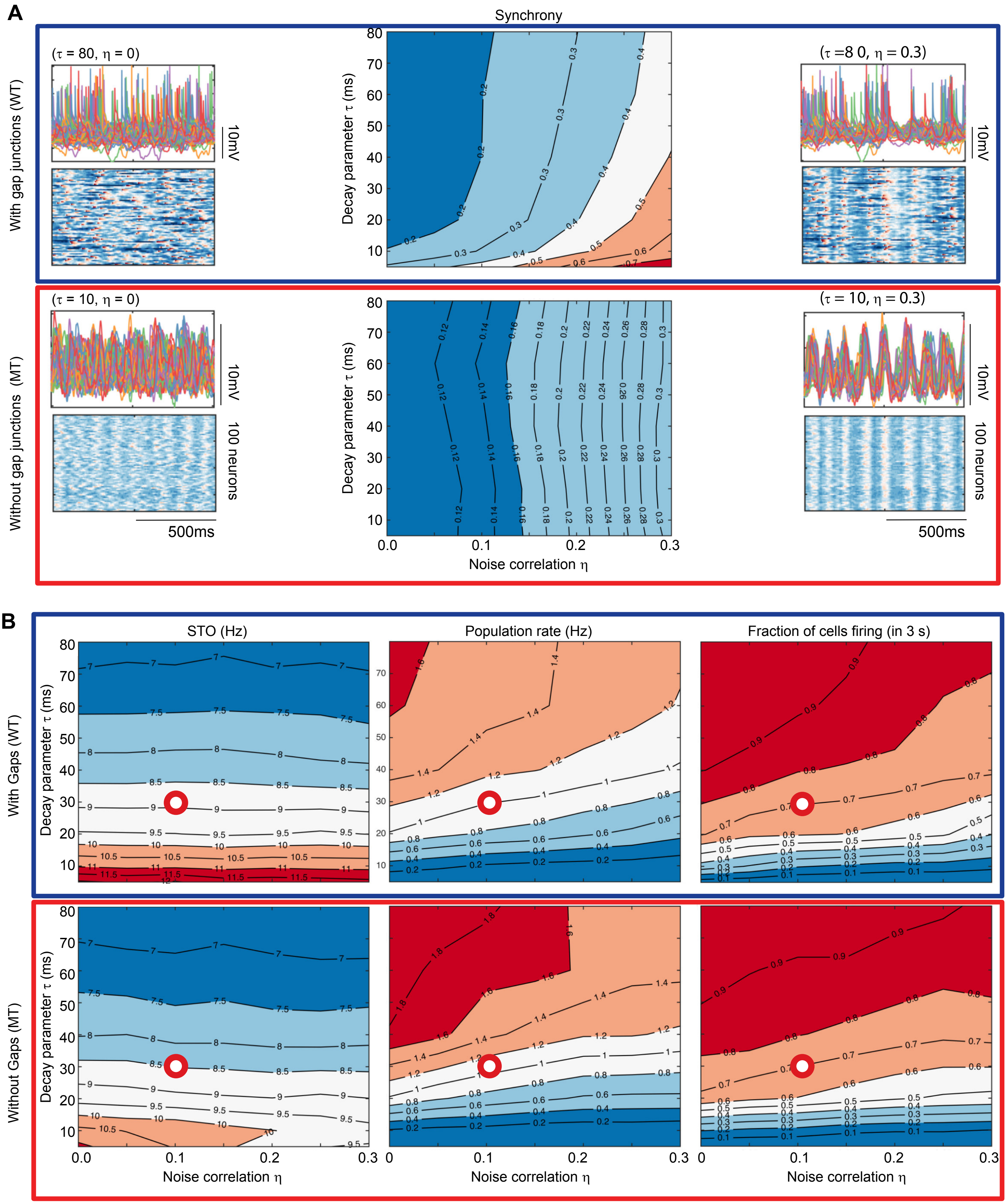

Fig. S5 | Behavior of model networks of identical composition as a function of the decay parameter of the Ornstein-Uhlenbeck process (τ) and the noise correlation (η). Model networks have been computed using a systematic exploration of the parameter space using 56 instances of the network model. The contour plots indicate isolines for synchrony (**A**), frequency of subthreshold oscillations (STOs), population firing rate and proportion of active cells (**B**). The results presented in the main text come from a model network with parameters chosen such as to display STOs with mean of 9 Hz, a population rate of about 1 Hz and with more than 70% of cells firing in three seconds (95% of cells fire within 10 s of simulation time). The position of this network in the contour plots is indicated with a red circle. In **A**, the thumbnails exemplify behaviors of extreme instances of the model network both as membrane potentials traces (top) and as heatmaps of the membrane potential (bottom). Arrows indicate parameter space coordinates of these examples. The decay of the Ornstein-Uhlenbeck process (τ) mostly impacts the firing rate of the model networks, while noise correlation (η) has a direct effect on synchrony. (**B**) Gap junctions amplify the input correlation given to the neurons, while having a minor effect on other aspects of the network dynamics.
