# Supplementary material for: Quasiperiodic rhythms of the inferior olive
Source: PLoS Comput Biol. 2019 May 6;15(5):e1006475. doi: 10.1371/journal.pcbi.1006475 (PMC6538185; doi:10.1371/journal.pcbi.1006475)
Supplement: S6 Fig — (A) The Ca2+ T-type conductance is varied in the range of 0.5 to 1.1 mS/cm2, resulting in a range of oscillatory properties of the individual model cells. The left axis in the panels of the main diagonal display cell counts for the histograms. The right axis besides the rightmost panel displays the indicated continuous variable. The set of non-oscillating (zero amplitude, zero frequency) cells constituted about 25% of the model network in the absence of gap junctions (red), were engaged in the oscillation when gaps were added to these cells (blue). In the absence of contextual input the distribution of frequencies had peaks at zero and sharply synchronizes at approximately in the absence of Ornstein-Uhlenbeck input. (B) Comparison between the activity of networks with (WT) and without (MT) gap junctions under contextual input. MT cells have received compensation in the leak conductance of the cell. A slight increase of firing rate for the MT can be observed, and also a narrower distribution of frequencies, in comparison with the noiseless case. (PDF) [file pcbi.1006475.s006.pdf]

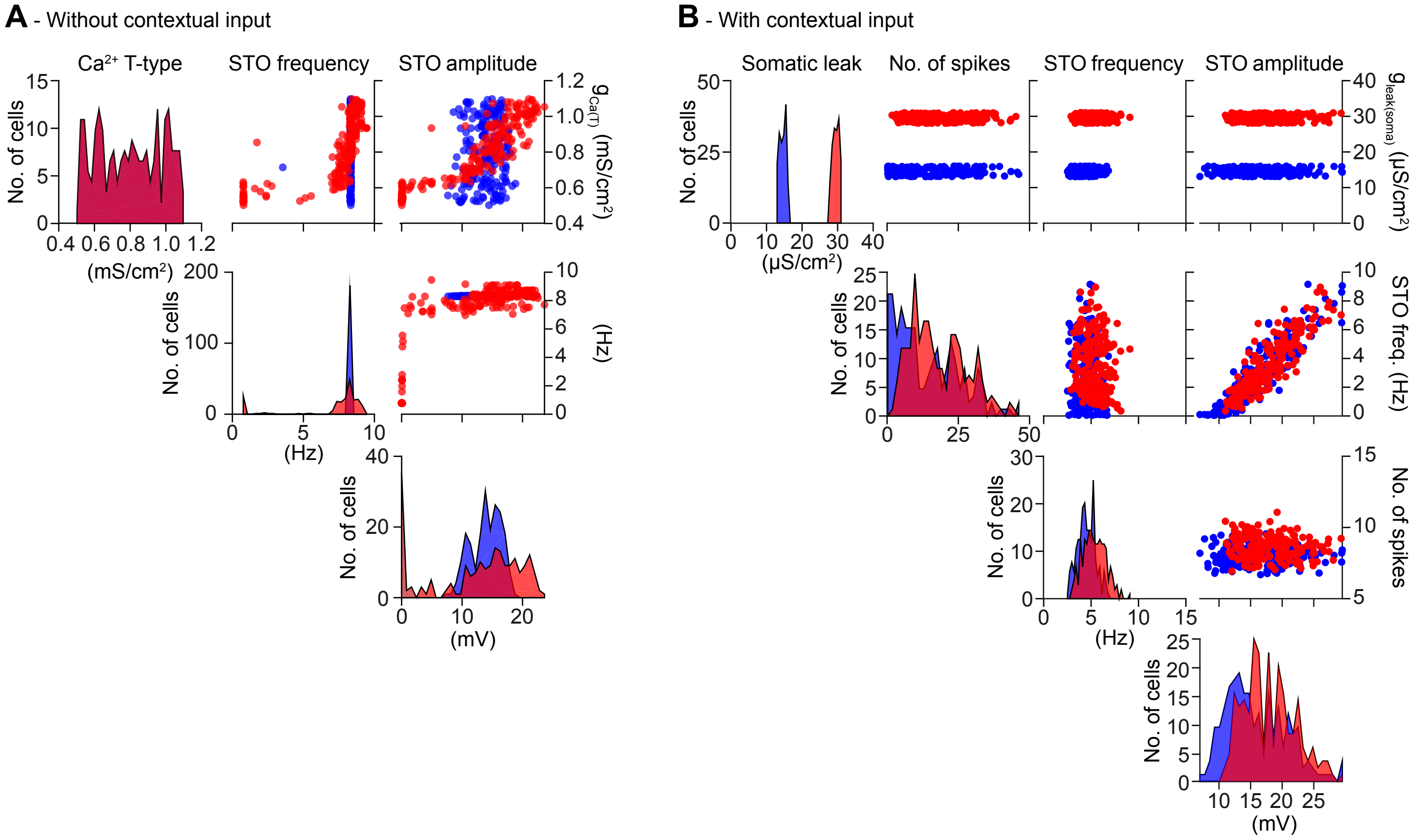

Fig. S6 | Physiological properties of the individual model cells as a function of Ca^{2+} T-type conductance in the absence of contextual input in the presence and absence of gap junctional coupling. (A) The Ca^{2+} T-type conductance is varied in the range of 0.5 to 1.1 mS/cm^2, resulting in a range of oscillatory properties of the individual model cells. The left axis in the panels of the main diagonal display cell counts for the histograms. The right axis besides the rightmost panel displays the indicated continuous variable. The set of non-oscillating (zero amplitude, zero frequency) cells constituted about 25% of the model network in the absence of gap junctions (red), were engaged in the oscillation when gaps were added to these cells (blue). In the absence of contextual input the distribution of frequencies had peaks at zero and sharply synchronizes at approximately in the absence of Ornstein-Uhlenbeck input. **(B)** Comparison between the activity of networks with (WT) and without (MT) gap junctions under contextual input. MT cells have received compensation in the leak conductance of the cell. A slight increase of firing rate for the MT can be observed, and also a narrower distribution of frequencies, in comparison with the noiseless case.
