# Supplementary material for: Quasiperiodic rhythms of the inferior olive
Source: PLoS Comput Biol. 2019 May 6;15(5):e1006475. doi: 10.1371/journal.pcbi.1006475 (PMC6538185; doi:10.1371/journal.pcbi.1006475)
Supplement: S7 Fig — The post-spiking behavior of model neurons after a depolarization was examined for a grid of parameters, within known experimental values. A depolarizing current pulse triggers spikes at 100 ms. The rebound behavior is dependent on a complex interplay between a number of parameters, but most importantly, it exists in a rather narrow range of the parameter space between T- and P/Q-type Ca2+ channels. For very low levels of Cav3.1 (T-type) expression, the model cell settles on an unphysiologically saturated depolarization after spiking. The cells in our model have Cav2.1 (P/Q-type) expression around 4.5 and Cav3.1 between 0.6 and 1.1. (PDF) [file pcbi.1006475.s007.pdf]

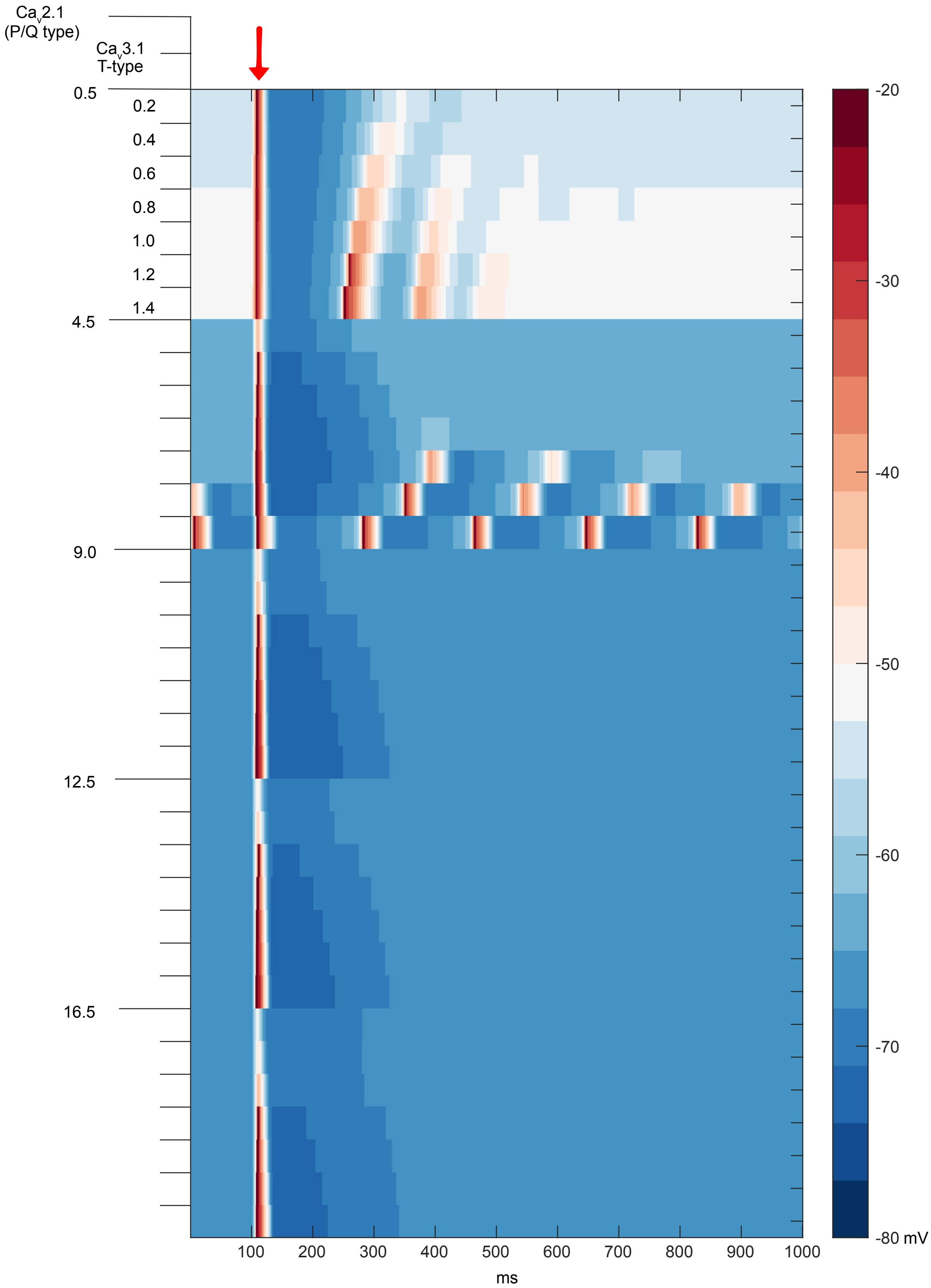

Fig. S7 | Rebound firing occurs only in a small region of parameter space. The post-spiking behavior of model neurons after a depolarization was examined for a grid of parameters, within known experimental values. A depolarizing current pulse triggers spikes at 100 ms. The rebound behavior is dependent on a complex interplay between a number of parameters, but most importantly, it exists in a rather narrow range of the parameter space between T- and P/Q-type Ca^{2+} channels. For very low levels of Ca$_v$3.1 (T-type) expression, the model cell settles on an unphysiologically saturated depolarization after spiking. The cells in our model have Ca$_v$2.1 (P/Q-type) expression around 4.5 and Ca$_v$3.1 between 0.6 and 1.1.
