# Supplementary material for: Quasiperiodic rhythms of the inferior olive
Source: PLoS Comput Biol. 2019 May 6;15(5):e1006475. doi: 10.1371/journal.pcbi.1006475 (PMC6538185; doi:10.1371/journal.pcbi.1006475)
Supplement: S8 Fig — (A) In order to test the impact of the presumed phase of the inferior olivary neurons in vivo, we applied a “gallop” stimulation pattern, alternating short (250 ms) and long (400 ms) intervals. Air puffs (vertical bars) were delivered to the whisker pad. Complex spikes were counted in response windows (RW) 20–200 ms post-stimulus and cells were sorted as a function of the ratio between the numbers of complex spikes in short and long intervals (indicated as horizontal dash between filled and empty bars). In this analysis, we included all RWs, irrespective of whether the preceding RW contained a complex spike or not (cf. Fig 10). For each Purkinje cell the relative response probabilities for the long and short intervals are illustrated as the length of the filled and open bars, respectively. None of the Purkinje cells showed a significant difference in the response probability between the two intervals (all p > 0.05 on Fisher’s exact test). (B) Comparison of the response biases between all trials (”All”, A) and only those trials that followed an RW with at least one complex spike (”Cond”, Fig 10A). Shown are the 25 and 75% quantiles and median per population. (PDF) [file pcbi.1006475.s008.pdf]

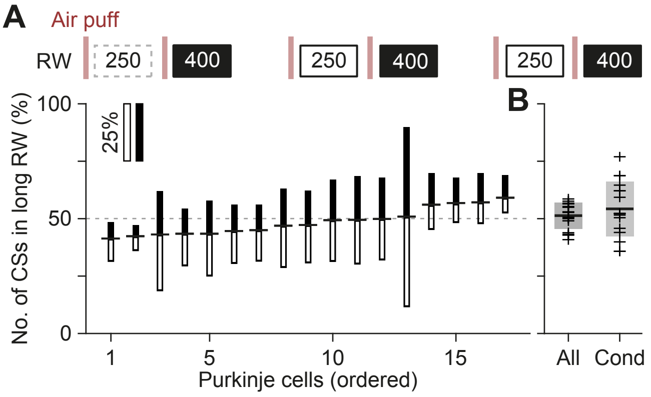

Fig. S8 | No phase-dependent spiking probabilities observed *in vivo*.
(A) In order to test the impact of the presumed phase of the inferior olivary neurons *in vivo*, we applied a “gallop” stimulation pattern, alternating short (250 ms) and long (400 ms) intervals. Air puffs (vertical bars) were delivered to the whisker pad. Complex spikes were counted in response windows (RW) 20-200 ms post-stimulus and cells were sorted as a function of the ratio between the numbers of complex spikes in short and long intervals (indicated as horizontal dash between filled and empty bars). In this analysis, we included all RWs, irrespective of whether the preceding RW contained a complex spike or not (cf. Fig. 10). For each Purkinje cell the relative response probabilities for the long and short intervals are illustrated as the length of the filled and open bars, respectively. None of the Purkinje cells showed a significant difference in the response probability between the two intervals (all $p > 0.05$ on Fisher’s exact test). **(B)** Comparison of the response biases between all trials (”All”, **A**) and only those trials that followed an RW with at least one complex spike (”Cond”, Fig. 10A). Shown are the 25 and 75 % quantiles and median per population.
